# EAACI Task force Clinical epidemiology of anaphylaxis: experts’ perspective on the use of adrenaline autoinjectors in Europe

**DOI:** 10.1186/s13601-020-00317-y

**Published:** 2020-05-11

**Authors:** Magdalena Kraft, Sabine Dölle-Bierke, Paul J. Turner, Antonella Muraro, Montserrat Fernández-Rivas, Linus Grabenhenrich, Margitta Worm

**Affiliations:** 1 Division of Allergy and Immunology, Dpt. of Dermatology, Venerology and Allergology, Charité – Universitätsmedizin Berlin Corporate Member of Freie Universität Berlin, Humboldt-Universität zu Berlin, and Berlin Institute of Health, Berlin, Germany; 2 Section of Inflammation, Repair & Development, National Heart & Lung Institute, Imperial College London, London, UK; 3 Food Allergy Referral Centre, Department of Women and Child Health, Padua General University Hospital, Padua, Italy; 4 Department of Allergy, Hospital Clinico San Carlos, Universidad Complutense, IdISSC, ARADyAL, Madrid, Spain; 5 Department for Infectious Disease Epidemiology, Robert Koch-Institut, Berlin, Germany

**Keywords:** Anaphylaxis, Anaphylactic reaction, Adrenaline autoinjector, AAI, Epinephrine autoinjector, European Academy of Allergy and Clinical Immunology guidelines, European Medical Agency

## Abstract

**Background:**

Worldwide, guidelines recommend the use of adrenaline autoinjectors (AAIs) for self-medication in patients who experience severe allergic reaction. The European Medical Agency recommends the prescription of two AAIs, which should be carried by patients at all times. The European Academy of Allergy and Clinical Immunology guidelines propose to prescribe a second AAI under some defined conditions. In the present study, we aimed to examine the adherence to these guidelines and prescription behavior of allergy experts regarding the number of AAIs prescribed for a given patient.

**Methods:**

A standardized questionnaire was applied to the participants of the 5th International Conference of the Network of Online Registration for Anaphylaxis (NORA e. V.). Twenty-six experts (medical doctors with at least 2 years of experience in the field of anaphylaxis) answered the questions regarding the number of autoinjectors prescribed and the reasons influencing their decisions.

**Results:**

Sixty-eight percent of the experts usually prescribed one AAI, while 32% prescribed two. The pediatricians and physicians with less experience tended to prescribe two autoinjectors more frequently. The experts were more likely to prescribe two adrenaline autoinjectors if the patient was a child, had a previous severe reaction, had mastocytosis, asthma, cardiovascular disease, or high body weight, or lived far from the emergency department.

**Conclusion:**

Our data confirm the lack of consensus regarding the number of AAIs to prescribe. Despite the European Medical Agency recommendation, the majority of allergy experts prescribed one autoinjector per patient. However, under distinct circumstances (e.g. mastocytosis, asthma, excess body weight, a history of severe anaphylaxis, or restricted access to immediate emergency), experts tended to prescribe more AAIs, which is in accordance with the European Academy of Allergy and Clinical Immunology guidelines.
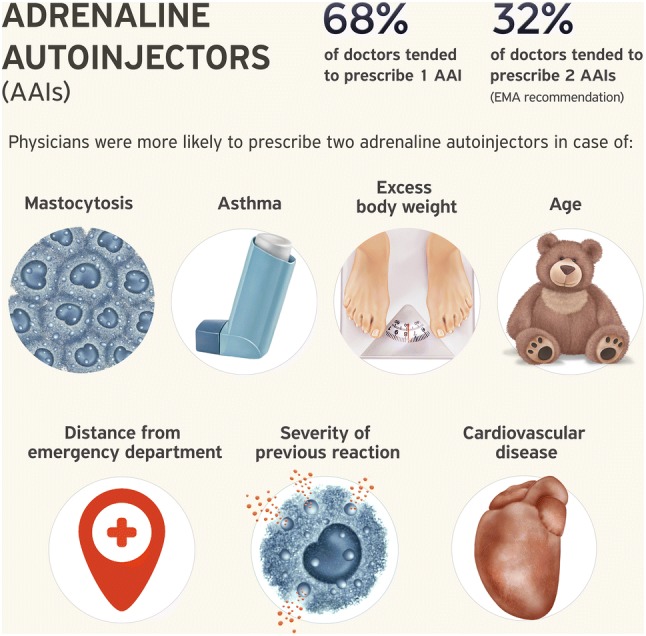

## Background

Globally, guidelines recommend the use of adrenaline autoinjectors (AAIs) for self-medication in patients who experience severe allergic reaction [1–3]. However, previous data from the European Anaphylaxis Registry (Network of Online Registration for Anaphylaxis (NORA e. V.)) has shown that the use of AAI by patients (or their carers) is low, and remains low despite the implementation of educational initiatives [4]. The European Medical Agency (EMA) recommends the prescription of two AAIs, which should be carried by patients at all times [5]. This is also reflected in existing European guidelines, where specific scenarios are listed when prescribers should consider the need to provide a second AAI [1]. Such factors include coexisting unstable or moderate-to-severe persistent asthma and a food allergy, mast cell diseases and elevated baseline tryptase, lack of access to emergency medical services, previous reaction requiring more than one dose of adrenaline, previous near fatal anaphylaxis, and lastly, if the available AAI dose is too low with respect to body weight. These recommendations are based on data which have shown that a single dose of adrenaline is not always sufficient to control an anaphylaxis [1, 2, 6–8]. However, the evidence grade for these recommendations is low (IV or V).

In the present study we performed a questionnaire-based analysis of the AAI prescription behavior among participants of the 5th International Conference of NORA e. V. We sought to investigate how experts from specialized tertiary allergy centers vary in their daily practice regarding the number of AAI prescribed, and which factors influence their decisions.

## Methods

**Fig. 1:**
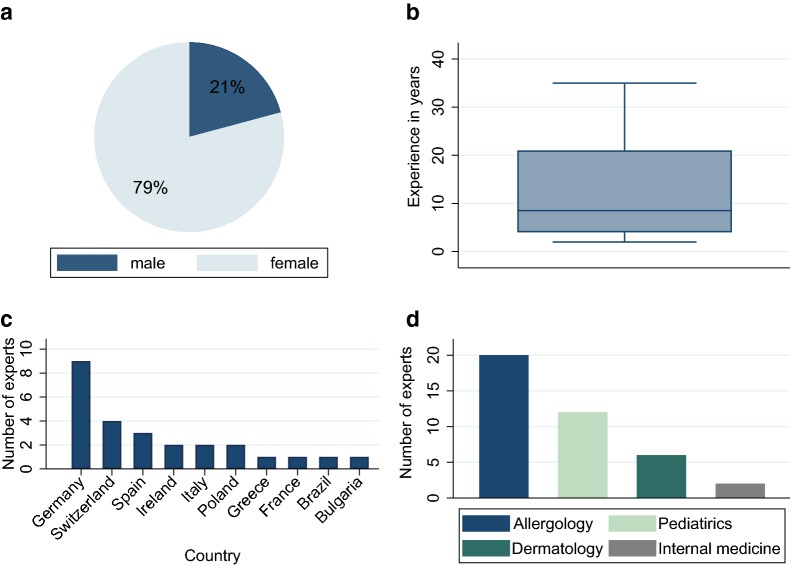
Demographic characteristic of the study population regarding gender (**a**), experience in the field of anaphylaxis (**b**), country (**c**) and medical specialty (**d**)

Allergy centers throughout Europe can participate in the NORA e. V. and contribute to the Anaphylaxis Registry. The biennial international conference is held to exchange knowledge and data gained within and outside the network regarding anaphylaxis. During the conference the European Academy of Allergy and Clinical Immunology (EAACI) task force on the implementation of guidelines in anaphylaxis is having a meeting. The 5th International Conference was held in April 2019. Forty-five experts participated in the conference; data was collected from 26 attendees who were medical doctors with at least 2 years of experience in the field of anaphylaxis using a questionnaire (Additional file 1). The demographic data of this study population is presented in Fig. 1. Seventy-nine percent of the experts were females (n = 19, Fig. 1a). The median experience in the field of anaphylaxis was 8.5 years (Fig. 1b). Attendees came from 10 different countries (Fig. 1c), and represented a range of medical specialties (Fig. 1d, multiple selection possible) including allergology and pediatric allergology (n = 20), pediatrics (n = 12), dermatology (n = 6), and internal medicine (n = 2). The data were analyzed using STATA^®^ 15.0 statistical software (Stata Corp., College Station, Texas).

## Results

### AAI prescription behavior among allergists from NORA

**Fig. 2:**
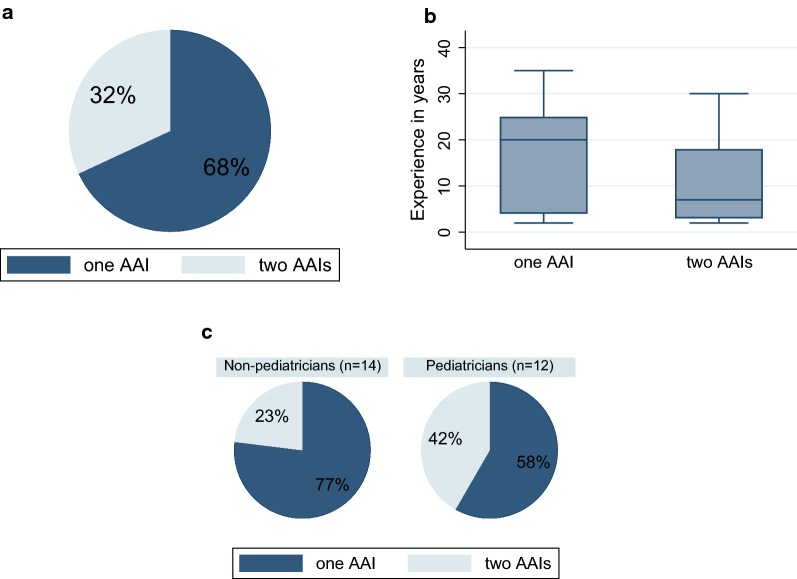
Proportion of experts (n = 26) who prescribe usually one or two adrenaline autoinjectors per one patient in total (**a**) and separate for pediatricians and non-pediatricians (**c**). The distribution of experience years in the field of anaphylaxis in the group of experts who usually prescribe one and in the group who usually prescribe two autoinjectors (**b**)

Sixty-eight percent of the experts (n = 17) described their usual practice as prescribing one AAI and 32% (n = 9) prescribed two AAIs (Fig. 2a) in usual. The younger physicians and pediatricians tended to prescribe more frequently two autoinjectors for one patient (Fig. 2b, c).

### Factors influencing the AAI prescription behavior

**Fig. 3:**
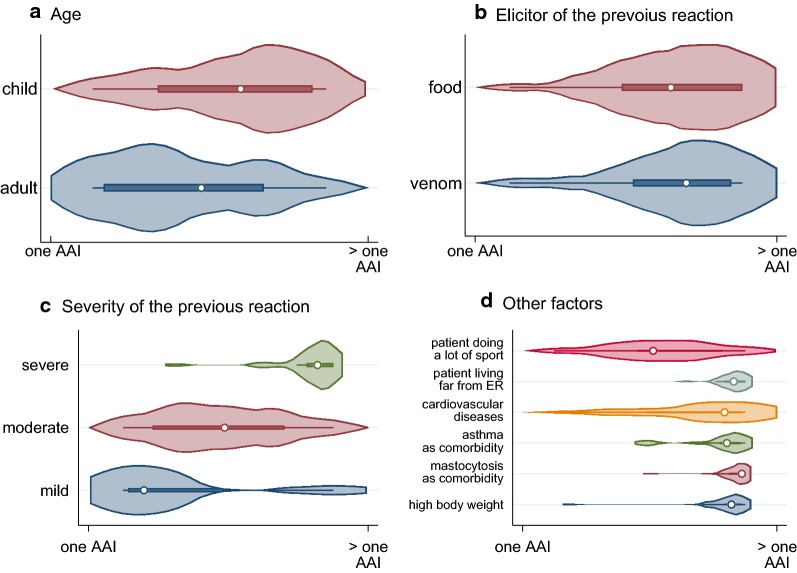
Factors influencing the AAI prescription behavior. The violin plots indicate if the given factor: age (**a**), elicitor (**b**), severity (**c**) or other factors (**d**) would influence the experts to prescribe one (left end of the scale) or more than one (right end of the scale) autoinjectors

To investigate factors which might influence prescription decisions, participants were asked to complete on a visual analogue scale the degree to which a given factor might influence their decision to prescribe one (left end of the visual scale) or more than one (right end of the visual scale) AAI. Figure 3 summarizes the results in form of violin plots for each item. Respondents tended to prescribe > 1 AAI if the patient was a child (Fig. 3a) or had a history of prior severe anaphylaxis reaction (Fig. 3c). Elicitors (food vs. insect) was not an important factor (Fig. 3b), while mastocytosis, asthma or cardiovascular diseases as comorbidity was. In addition, if the patient lived far from the emergency department, s/he would be more likely to be prescribed two AAIs (Fig. 3d).

**Fig. 4:**
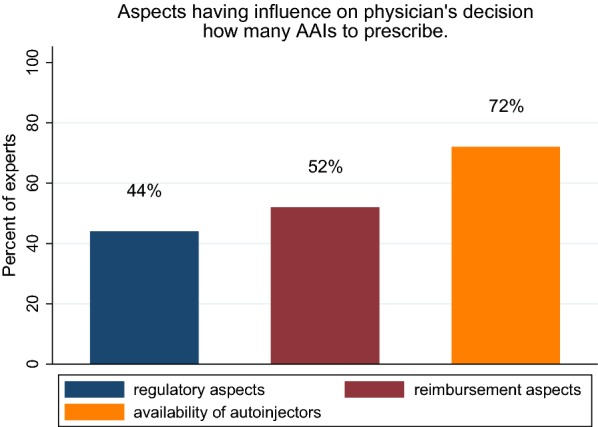
Proportions of experts being influenced by regulatory, reimbursement and availability aspects in their decision regarding number of AAIs per one patient

We then asked respondents as to whether their decision was influenced by legal and/or economic considerations (Fig. 4). Forty-four percent of them (n = 11) responded that their decisions were influenced by regulatory aspects, 52% (n = 13) by reimbursement issues, and 72% by the availability of AAI in the community. However, when asked if the shortage of AAIs in the previous months (this survey was performed in April 2019) had an impact on their prescription habits, most of the experts (58%, n = 16) denied this (data not shown).

## Discussion

Limited data exists regarding the implementation of guidelines on the prescription of AAI in Europe. Recent data from the EAACI revealed that only 59% of the allergists who answered a single questionnaire were following these recommendations (unpublished data). Data from the Anaphylaxis Registry show that 35% of patients with a history of anaphylaxis treated in tertiary allergy centers were prescribed more than one AAI [9]. Our data confirm the lack of consensus regarding the number of AAIs to prescribe, although following the EAACI guidelines [1], experts tended to prescribe more AAIs if the patient had mastocytosis, asthma, excess body weight, a history of severe anaphylaxis, or restricted access to immediate emergency treatment (Fig. 3). Children were more frequently prescribed > 1 AAI, which is consistent with data form the Anaphylaxis Registry in which ~ 50% of children were prescribed > 1 AAI compared with ~ 30% of adults [9]. There are significant geographical factors involved, as up to 92% children are prescribed 2 + AAIs in the UK, although this is likely to be a combination of children being prescribed one AAI for personal use and a separate AAI for school [10]. An additional factor could be the strong recommendation by the UK regulator to prescribe 2 AAIs (which are free in the UK to children) to be carried at all times.

We only partially examined the reasons behind experts’ decision to prescribe a single AAI. The regulatory and reimbursement aspects appear to play a role in approximately half of the experts. Other key barriers may be: high cost of devices, doubts regarding patients’ compliance to carry and use two AAIs, the belief that the second adrenaline dose will not be required, or a low evidence grade for the recommendation in the guidelines. For these reasons, we conclude that, measures to lower the costs of AAIs and more data supporting EMA recommendations are needed to increase the rate of patients being prescribed a second adrenaline dose.

The main limitation of our analysis is the small survey size. The survey was performed during the specialized allergology conference which took place in Germany and was associated with *EAACI Task force Clinical epidemiology of anaphylaxis* meeting, so the German experts and members of the Task force were overrepresented which may have biased the results. The second important limitation is the fact, that only a few questions examining the reasons for experts’ prescription habits were asked, which makes a more in-depth analysis of that important question not possible.

## Conclusions

Our data point out discrepancies even among specialists and indicate a need for a larger, representative survey among physicians form different European countries and wider medical specialties treating anaphylaxis patients. More detailed questions on the training and experience in the field of anaphylaxis and reasons for decision making should be asked. EAACI and other medical societies could be used as a platform to reach the physicians. The results of this survey could provide an important perspective which should be taken into consideration during guidelines development, decision making by regulatory health agencies and the development of standardized trainings for young physicians.

## Supplementary information

**Additional file 1.** Questionnaire.

## Data Availability

The dataset generated and analyzed during the current study are not publicly available due to data protection but are available from the corresponding author on reasonable request (excluding personal data).

## Abbreviations

AAI: Adrenaline autoinjector
EAACI: European Academy of Allergy and Clinical Immunology
EMA: European Medical Agency
NORA: Network of Online Registration for Anaphylaxis
