# Supplementary material for: EAACI Task force Clinical epidemiology of anaphylaxis: experts’ perspective on the use of adrenaline autoinjectors in Europe
Source: Clin Transl Allergy. 2020 May 11;10:12. doi: 10.1186/s13601-020-00317-y (PMC7216364; doi:10.1186/s13601-020-00317-y)
Supplement: Supplementary file 1 — Additional file 1. Questionnaire. [file 13601_2020_317_MOESM1_ESM.pdf]

NETWORK FOR ONLINE-REGISTRATION OF ANAPHYLAXIS

Expert habits on the prescription of adrenaline autoinjectors

Please grade the severity **of each single factor** on a visual analog scale **by marking a vertical line** on a provided horizontal scale. Please consider **how likely a given factor would influence your decision to prescribe one or more than one autoinjector.** Example:

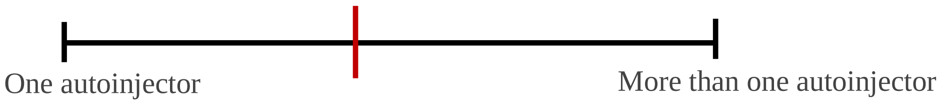

How many adrenaline autoinjector do you usually prescribe for one patient?

☐ one

☐ two

☐ three

☐ four

☐ more than four

What factors influence your decision to prescribe more than one adrenaline autoinjector to an individual patient?

	One AAI	> one AAI
gender: male		
gender: female		
age: child		
age: adult		
elicitor: venom		
elicitor: food		
elicitor : drug		

Thank you very much for taking part in this survey!

	**One AAI**	**> one AAI**
high body weight		
previous reaction: mild		
previous reaction: moderate		
previous reaction: severe		
mastocytosis as comorbidity		
asthma as comorbidity		
cardiovascular diseases		
patient living far form emergency department		
patient doing a lot of sports		
other life style factors:		

(please specify: ______________________________)

Dose following aspects influence your decision:

regulatory aspects	☐ Yes	☐ No
reimbursement aspects	☐ Yes	☐ No
availability of autoinjectors	☐ Yes	☐ No

Did the shortages of the autoinjectors in the last months influence your prescription habits?

☐ Yes ☐ No

NETWORK FOR ONLINE-REGISTRATION OF ANAPHYLAXIS

Expert opinion on the severity of anaphylaxis symptoms

Grading of anaphylaxis severity requires an expert-driven consensus. Therefore, we are kindly asking you to fill out the following survey.

Please grade the severity **of each single symptom** on a visual analog scale **by marking a vertical line** on a provided horizontal scale. Please do not consider subjective discomfort of a given symptom for a patient but rather **how likely a given symptom represents a severe reaction.** Example:

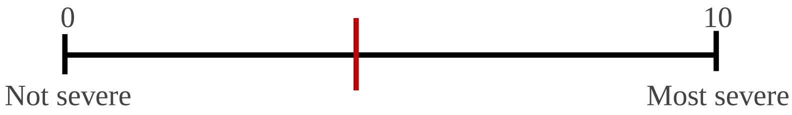

General information:

1. In which country is your centre located? ______________________________

2. What is your medical specialty?
More than one answer is possible.
☐ Dermatology
☐ Paediatrics
☐ Pneumology
☐ Allergology (incl. paediatric allergology)
☐ Other (please specify: ______________________________)

3. How long is your experience in the field of allergy/anaphylaxis? ____________

4. Please indicate you name here: ______________________________

please turn →

angioedema
laryngeal edema
erythema/ flush
pruritus/ itch
urticaria
conjunctivitis
abdominal pain/ cramps
abdominal distension
diarrhoea
dysphagia
vomiting
incontinence
nausea
respiratory arrest
dyspnea/ shortness of breath
chest tightness
cough
change in voice
throat tightness
wheezing (expiratory)
rhinitis
stridor (inspiratory)
loss of consciousness
hypotension / collapse
chest pain / angina
palpitations / cardiac arrhythmia
cardiac arrest
dizziness
tachycardia
reduction of alertness
dysphonia
dysarthria
hotness/ sweating / trembling
tingling/burning of hands/feet, paresthesia
sight disorder
agonizing pain
cyanosis
pallor
death

Thank you very much for taking part in this survey!
